# Reverse Transcription Cross-Priming Amplification–Nucleic Acid Test Strip for Rapid Detection of Porcine Epidemic Diarrhea Virus

**DOI:** 10.1038/srep24702

**Published:** 2016-04-19

**Authors:** Feng-Xue Wang, Dan-Yi Yuan, Ya-Nan Jin, Lin Hu, Zhi-Yong Sun, Qian He, Shi-Hua Zhao, Shu-Bai Zhan, Yong-Jun Wen

**Affiliations:** 1 State Key Laboratory of Special Economic Animal Molecular Biology, Institute of Special Economic Animal and Plant Sciences, Chinese Academy of Agricultural Sciences, No. 4899 Juye Avenue, Jingyue Economic and Technological Development Zone, Changchun, Jilin, 130112, People’s Republic of China; 2 Veterinary Research Institute, Inner Mongolia Academy of Agricultural and Animal Husbandry Sciences, Huhhot, Inner Mongolia, 010031, People’s Republic of China; 3 (Sino-USA) SiChuan Nabii Bio-Tech Co., Ltd., Chengdu, SiChuan, 610041, People’s Republic of China; 4 Ustar Biotechnologies (Hangzhou), Ltd., Hangzhou, Zhejiang, 310012, People’s Republic of China

## Abstract

Porcine epidemic diarrhea virus (PEDV) is a highly transmissible coronavirus that causes a severe enteric disease particularly in neonatal piglets. In this study, a rapid method for detecting PEDV was developed based on cross-priming amplification and nucleic acid test strip(CPA-NATS). Five primers specific for the N gene sequence of PEDV were used for the cross-priming amplification. Detection of amplification products based on labeled probe primers was conducted with strip binding antibody of labeled markers. The CPA method was evaluated and compared with a PCR method. The reverse transcription CPA system was further optimized for detecting PEDV RNA in clinical specimens. Results showed that the method was highly specific for the detection of PEDV, and had the same sensitivity as PCR, with detection limit of 10^−6^ diluted plasmid containing the target gene of PEDV. It was also successfully applied to detecting PEDV in clinical specimens. The reverse transcription CPA-NATS detection system established in this study offers a specific, sensitive, rapid, and simple detection tool for screening PEDV, which can contribute to strategies in the effective control of PEDV in swine.

Porcine epidemic diarrhea (PED) is an acute and highly contagious enteric disease characterized by severe enteritis, vomiting, and watery diarrhea in swine. The disease is caused by porcine epidemic diarrhea virus (PEDV), a member of the family Coronaviridae. Outbreaks of PED have been reported in many countries and caused a significant animal health problem and financial burden to the swine industry^1^,^2^,^3^,^4^,^5^. Since PED has a mobility of as high as 100% and a mortality of 80–100% in piglets less than 10 days old^6^, rapid and accurate diagnostic methods of PEDV are essential for effective control of this devastating disease in swine.

The genome of PEDV is approximately 28 kb nucleotides in length. At least seven open reading frames (ORFs) have been identified in the PEDV genome, with a characteristic gene order 5′-replicase (1a/1b)-S-ORF3-E-M-N-3′. Several methods including separation identification, serological method, PCR, and colloidal gold method have been used to detect PEDV. The traditional viral separation method has high specificity and sensitivity, but is time consuming and takes at least three to five days. The serological method is mainly used for the identification of PEDV in serum and not directly for fecal samples. The PCR method has high sensitivity, but requires special equipment and reagents that are unavailable at farm level. Although rapid detection through the immune colloidal gold technique has no requirements for a laboratory setting, its positive predictive value is low. The technology still needs bacterial culture as the ultimate standard. Thus, we applied the cross-priming amplification (CPA), which is a novel isothermal amplification reaction technology using Bst DNA polymerase, and multiple primers and probes, one or more of which is a cross primer. Five primers specific for the N gene of PEDV were used. The cross primer with a 5′tail encodes the complementary strand primer^7^. The amplification consisted of three steps: (1) extension and displacement with cross primer, (2) multiple extensions and displacements with multiple primer binding sites, and (3) further primer extension by primers with stabilized hairpin-like structures that form and act as templates leading to the target products. Then, the products labeled with FAM (fluorescein dye) and biotin were detected using a strip covered with the antibody against FAM or biotin^7^. Two red bands indicate a PEDV-positive result, whereas one red band indicates a PEDV-negative result.

This method is able to amplify approximately four bacterial cells in less than an hour with a high degree of specificity^7^. It does not require an initial denaturation step or a nicking enzyme and has a high specificity and sensitivity. The amplification is conducted under a constant temperature and combined with dipstick nucleic acid detection without electrophoresis. The system offers a relatively quick and easy method for field diagnostic testing of pathogens.

## Results

### Optimization of primer combinations

**Figure 1:**
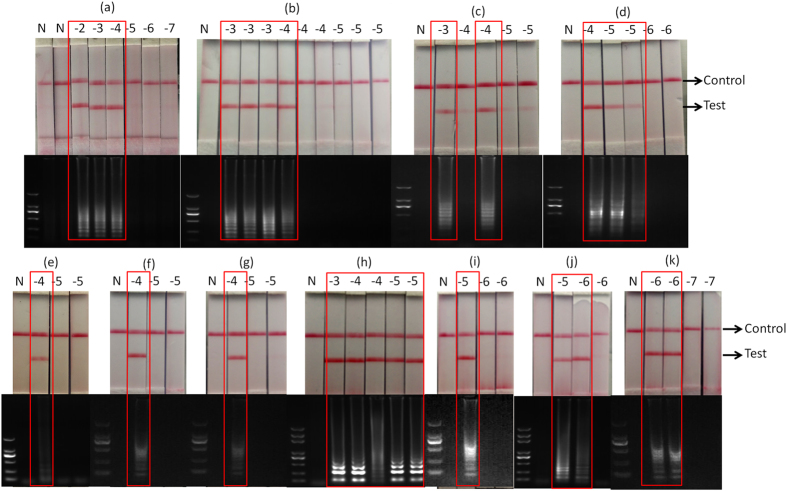
CPA-NATS and gel electrophoresis results of different components and conditions optimization: (**a**) external primers, (**b**) probe, (**c**) cross primer, (**d**) dNTP, (**e**) MgSO_4_, (**f**) betaine, (**g**) Bst DNA polymerase, (**h**) reaction time of 60 min, (**i**) reaction time of 75 min, (**j**) reaction time of 90 min, and (**k**) agarose gel electrophoresis confirmations of the CPA reaction corresponding to the results on the strips. N, negative control.

The optimization of amplification involved primer combinations with a high sensitivity and no false positive. A set of primers suitable for CPA detection of PEDV were designated by CPR-2, DF5B1, DF5F-12, F3-1, and B3-1 (Table 1). DF5F-12 and DF5B1 were labeled with FAM and biotin, respectively. Primer combination, CPR-2, DF5B1, DF5F-12, F3-1, and B3-1 gave a high sensitivity with no false positive (Fig. 1a up). Gel electrophoresis results below the strip graph confirmed the CPA reaction corresponding to the results on the strip (Fig. 1a down).

### Optimization of CPA assay

Optimization of CPA assay included concentration optimizations of external primers, probe and cross primer. Using concentrations ranging from 0.1 μL to 0.6 μL, the optimal concentrations of external primers were determined to be 0.1 μL of 20 μM F3-1 and 0.2 μL of 20 μM B3-1 (Fig. 1b).

Probes were optimized by testing concentrations (from 0.2 μL to 0.8 μL) of one probe against a fixed concentration of another probe. The optimal concentrations were 0.3 μL and 0.6μL for 20 μM DF5F-12 and 20 μM DF5B1, respectively (Fig. 1c).

The optimal concentration of the cross primer was 0.4 μL of 20 μM CPR-2 after testing with concentrations ranging from 0.1 μL to 1 μL. (Fig. 1d). Other reagents for the assay including MgSO_4_, betaine, deoxy-ribonucleoside triphosphate (dNTP), and Bst DNA polymerase were also optimized. The final volumes of dNTP (10 mM), MgSO_4_ (50 mM), betaine (5 M), and Bst DNA polymerase (10 U/μL) were 0.8, 0.4, 6, and 1 μL, respectively (Fig. 1e–h). A reaction temperature of 63 °C was selected for the CPA assays, similar to previous report^8^. The reaction time was extended by 15 and 30 min on the basis of the optimum experimental conditions to determine the relationship between reaction time and sensitivity. Our results indicated that, when CPA reactions were incubated for 90 min, favorable results were obtained without nonspecific amplification (Fig. 1k). The sensitivity of CPA was higher than that under 60 min (Fig. 1i) and 75 min (Fig. 1j). In addition, amplification results from the optimized CPA assay correlated 100% with those based on gel electrophoresis.

Finally, the total volume for the CAP amplification was 20 μL. The optimal concentration of each reagent was as follows: CPR-2, 0.4 μM; F3-1, 0.1 μM; B3-1(20 μM), 0.2 μM; DF5F(20 μM), 0.3 μM; DF5B(20 μM), 0.6 μM; MgSO_4_, 2 mM; betaine (5 M), 1.5 M; dNTP(10 mM), 0.8 μL; and 1 × Bst DNA polymerase buffer and Bst DNA polymerase (10 U/μL), 1 μL. The assay was able to detect plasmid containing PEDV target at 10^−4^ dilution after 60 min amplification and at 10^−6^ dilution after 90 min. No false positive was detected after 120 min incubation.

### Reverse transcriptioncross-priming amplification (RT CPA)

An assay combining RT and CPA was optimized to detect PEDV RNA from clinical specimens. AMV (BioFlux, China), Gsp Fast polymerase (Ustar, China), and RNA secure (Thermo Fisher, USA) in the RT CPA system were optimized. The optimal content of each component in the RT CPA system was as follows: CPR-2 (20 μM), 0.4 μM; F3-1 (20 μM), 0.1 μM; B3-1 (20 μM), 0.2 μM;DF5F (20 μM), 0.3 μM; DF5B(20 μM), 0.6 μM; MgSO_4_(50 mM),2 mM; betaine (5 M), 1.5 M; dNTP(10 mM), 0.08 μM; Gsp Fast polymerase (10 U/μL), 0.2 U/μL; RNA secure, 1 μL; and AMV (5 U/μL), 0.05 U/μL, 1 × Gsp Fast polymerase buffer, 2 μL. The total volume was 20 μL.

### Sensitivity and specificity of reverse transcription cross-priming amplification and nucleic acid test strip (RT CPA-NATS) system

**Figure 2:**
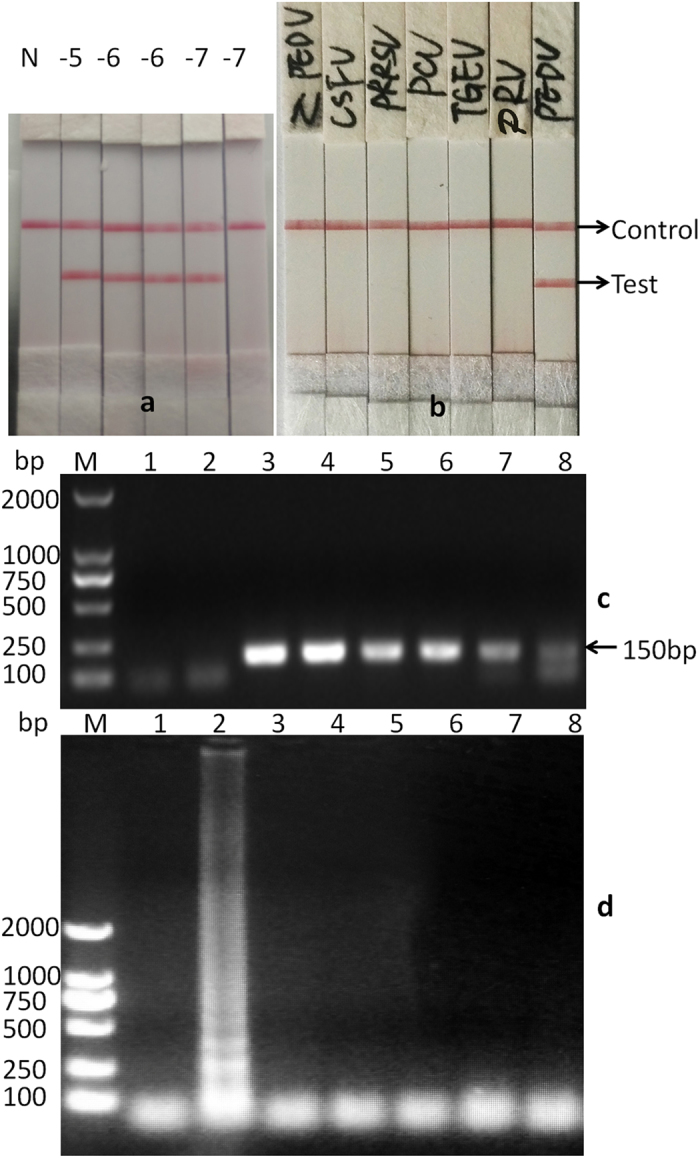
Sensitivity and specificity of RT CPA-NATS and RT-PCR: (**a**) sensitivity of CPA-NATS. N means negative control. −5, −6, −7 means 10^−5^, 10^−6^, 10^−7^ dilution. The detection limit was defined to 10^−6^ dilution, because 10^−7^ dilution plasmid was detected out randomly. (**b**) specificity of RT CPA-NATS. N: Negative control. The pathogens used to detect the specificity of RT-NATS and RT-PCR were CSFV, PRRSV, PCV, TGEV, PRV and PEDV. (**c**) sensitivity of RT-PCR. M: DNA Ladder DL2000. Lane 1 and 2 is negative control. Line 3–8 is 10^−1^~10^−6^. The detection limit was 10^−6^ dilution. The products size is about 150 bp. (**d**) gel electrophoresis confirmation of RT CPA-NATS. M: DNA Ladder DL2000. Lane 1: Negative control. Lane 2~Lane 8: PEDV, CSFV, PRRSV, PCV, TGEV, and PRV.

Ten times dilution series of plasmid was tested using CPA-NATS to determine its sensitivity. At the same time, reverse transcription PCR(RT-PCR) was used to confirm the CPA-NATS results. The results showed that 10^−6^ diluted plasmid could be detected consistently by CPA-NATS. However, results with 10^−7^ diluted plasmid were inconsistent (Fig. 2a). Thus, 10^−6^ was considered the detection limit of CPA-NATS, which had a similar sensitivity compared to that of RT-PCR (Fig. 2c).

The specificity of the RT CPA-NATS method for detecting PEDV was evaluated by testing other common pathogenic viruses of swine, including porcine circovirus virus (PCV), transmissible gastroenteritis virus (TGEV), porcine reproductive and respiratory syndrome virus (PRRSV), classical swine fever virus (CSFV), and porcine pseudorabies virus (PRV). Data showed 100% specificity against these viruses (Fig. 2b,d), comparable to RT-PCR.

### Evaluation and validation of RT CPA-NATS with clinical specimens

**Figure 3:**
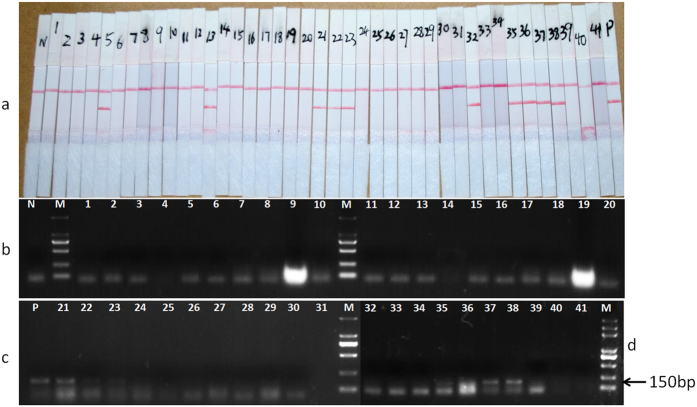
Evaluation of 41 clinical specimens from Chengdu, Harbin, and Jinan by RT CPA-NATS: (**a**) RT CPA-NATS data and (**b**,**c**) gel electrophoresis data. The product of RT-PCR is approximately150 bp. N, negative control; P, positive control. Samples 1–20 were obtained from Chengdu, 21–31 from Harbin, and 32–41 from Jinan. Seven samples (Nos 21, 22, 23, 29, 35, 37, and 38) were tested PEDV positive by RT-PCR. However, 10 samples (Nos 5, 13, 21, 22, 23, 32, 35, 36, 37, and 38) were tested PEDV positive by RT CPA-NATS.

A total of 41 fecal samples from swine suspected of PEDV infections collected in Chengdu, Harbin, and Jinan were tested for further evaluation and validation of the RT CPA-NATS in the detection of PEDV. Ten samples (No. 5, 13, 21, 22, 23, 32, 35, 36, 37, and 38) were detected positive by RT CPA-NATS (Fig. 3a). By contrast, only seven samples (No. 21, 22, 23, 29, 35, 37, and 38) were detected positive by RT-PCR (Fig. 3b–d). The test results of the clinical specimens by RT CPA-NATS and RT-PCR were also summarized in Table 2. The number of PEDV-positive samples detected by RT CPA-NATS was 2 of 20 in Chengdu, 3 of 11 in Harbin, and 5 of 10 in Jinan (Table 3). However, two samples (one from Chengdu and one from Jinan) were detected positive by RT CPA-NATS but negative by RT-PCR. Another sample from Harbin was detected positive by RT-PCR but negative by RT CPA-NATS.

**Figure 4:**
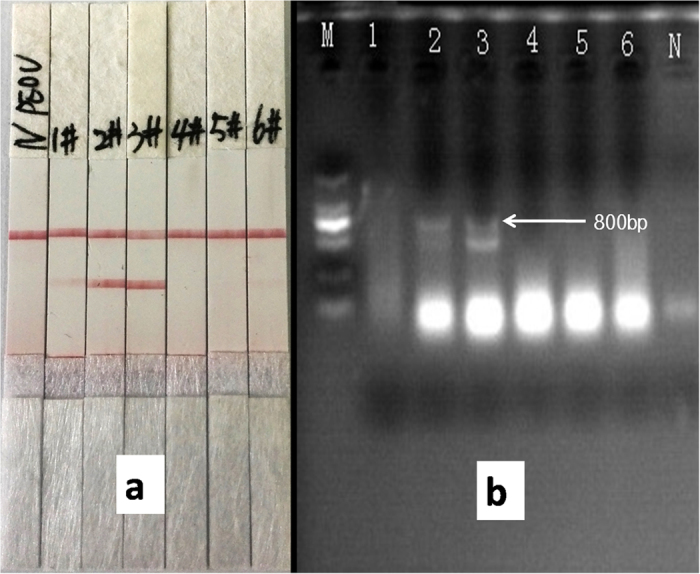
Trial of RT CPA-NATS detecting clinical specimens: (**a**) result of PEDV RT CPA-NATS and (**b**) result of PEDV RT-PCR.

Additionally, the system was validated by technicians with limited laboratory trainings on a pig farm. Six fecal swab specimens from sick piglets showing disease symptoms Suspected infected by PEDV were collected and tested for PEDV. Two of the six specimens (2# and 3#) were tested positive (Fig. 4a), which was in agreement with the results of RT-PCR (Fig. 4b).

### Ethics statement

The methods were carried out in accordance with Animal Epidemic Prevention Law of the People’s Republic of China. Animal experiments in this study were approved by the animal care and use committee of the Institute of Special Economic Animal and Plant Sciences and the animal welfare committee of the Jilin Province, China.

## Discussion

As one-step diagnostic system, RT CPA-NATS was capable of detecting PEDV in clinical specimens and displayed 100% specificity against several related porcine viruses. The CPA-NATS system based on an isothermal DNA amplification technology and does not require any sophisticated equipment. It has already been applied to clinical diagnosis of human infectious diseases such as tuberculosis^8^, where a double crossing CPA was used to amplify DNA of *Mycobacterium tuberculosis*. The present study employing the CPA-NATS technology was a single crossing CPA assay^7^, which is slightly different from a double crossing CPA. We successfully adopted the technology and optimize all reaction conditions to meet our objective in developing diagnostic system that combines reverse transcription and CPA for detecting RNA virus.

Several methods for the detection and diagnosis of PEDV have been described. Recently, a real-time reverse transcription loop-mediated isothermal amplification (RT-LAMP) method was reported to be easy to operate and simple to read results, and did not require sophisticated instruments^9^,^10^. However, RT-LAMP detects pathogens using reaction-turbidity as an indicator, which is sometimes difficult to determine without an instrument. The test strip result of CPA-NATS offers direct visualization by eyes, and is much easier to be interpreted. A variety of PCR methods have been developed to detect PEDV, such as a nanoparticle-assisted PCR assay^11^, duplex real-time RT-PCR^12^, and multiplex TaqMan probe-based real-time PCR^13^, which can detect and differentiate the variant and virulent strains of PEDV currently reported to be circulating in the US. The above mentioned molecular biology methods all need expensive instruments, which limits their clinical applications particularly in field laboratories or on farm.

The RT CPA-NATS system developed in the present study was capable of detecting PEDV in clinical specimens and distinguishing PEDV from other common viral pathogens causing pig diseases, namely, PCV, TGEV, CSFV, PRV, and PRRSV. It has a good specificity for detection of PEDV with the RT CPA-NATS methods. That is same as previous developed RT-PCR, real time RT-PCR or RT-LAMP^10^,^12^,^13^. By the contrast of RT CPA-NATS and RT-PCR, we found that RT CPA-NATS had a little better effect on detecting PEDV than RT-PCR and meanwhile it had a convenient and cheaper test procedure. In the previous research, ELISA and RT-PCR^14^, RT-PCR and real time RT-PCR^15^, RT-LAMP and real time RT-PCR^16^ were all compared with in virus detection. Different methods detecting the different viruses may show variable effect. Here, we have showed the comparison of RT CPA-NATS with RT-PCR on detecting PEDV. However, the system was incapable of discriminating variants and virulent strains of PEDV. Nevertheless, it offers an excellent tool for initial screening of clinical specimens for PEDV, and should make valuable contributions to the rapid identification of PEDV in order to prevent and control this devastating disease in swine.

In conclusion, our data showed that the RT CPA-NATS system developed for the detection of PEDV has significant advantages. The system was optimized and was comparable to RT-PCR that is the current recommended molecular method. Greater specificity and sensitivity, easy operation protocol and simple equipment make the RT CPA-NATS system ideal for the rapid detection of PEDV in clinical diagnosis.

## Methods

### Primers

Large fragment Bst DNA polymerase (Invitrogen, Beijing, China) was used in amplification reaction. According to the mechanism of CPA, the presence of a cross primer is essential^7^. The two pairs of outer primers, three upstream probes, four downstream probes, and three cross primers were designed for screening the best primer combinations. The primers that target the N gene of PEDV were designed based on a new PEDV sequence (KC189944.1, NT 26716–26873). The optimum primers and probes (Table 1) selected to develop CPA-NATS of PEDV were synthesized by Sangon Biotech (Shanghai, China). The probes were labeled with FAM or biotin.

### Plasmid as positive template

A constructed plasmid T-PEDV-N was used in developing CPA-NATS as positive template. Total RNA of PEDV was extracted with the E.A.N.A.™ Viral RNA Kit (OMIGA, Norcross, GA) as template to conduct RT-PCR to amplify the PEDV N gene fragment. The primers N-F/R were used to amplify PEDV N gene fragment (sequences were listed in Table 1). The amplicon was cloned into pMD18-T vector(Sangon Biotech, Shanghai, China) by TA cloning at site 425 and called T-PEDV-N. The concentration of T-PEDV-N was 241.5 μg/mL, with approximately 5.39 × 10^13^ copies/mL.

### CPA-NATS procedure

We conducted a probe hybridization reaction experiment and determined the selected primers that exhibited no reaction during probe hybridization. Subsequently, the CPA tests were conducted with different primer combinations. The initial test system was as follows: CPR(20 μM), 0.8 μL; F3(20 μM), 0.2 μL; B3(20 μM), 0.2 μL; DF5F(20 μM), 0.3 μL; DF5B(20 μM), 0.3 μL; Thermo Pol buffer (10×), 2 μL; MgSO_4_(100 mM), 0.4 μL; betaine (5 M), 2 μL; dNTP(10 mM), 0.8 μL; Bst DNA polymerase (10 U/μL), 1 μL; and template, 4 μL; sterile deionized water was added to achieved 20 μL. The products of CPA were diluted with1×saline sodium citrate buffer and dropped onto the sample pad of the test strips (Ustar Biotech, Hangzhou, China). The test strips were observed after 1 min. Positive PEDV is denoted by two red lines, with the top line for quality control and the bottom line for test. Only the control line is negative. Otherwise, only the test line is invalid.

### Specifity of CPA-NATS

We further evaluated the specificity of the CPA method for the detection of PEDV by testing other viruses, such as PCV, TGEV, PRV, and PRRSV, which cause similar symptoms in pigs. The genome (RNA or DNA) was extracted with the E.A.N.A.™ Viral RNA Kit (OMIGA, Norcross, GA) or E.A.N.A.™ Viral DNA Kit (OMIGA, Norcross, GA). The viruses were detected by the reverse transcription CPA-NATS (RT CPA-NATS) system.

### Optimization of RT CPA-NATS

In order to detect the PEDV RNA from clinical specimens, a system including RT and CPA was optimized. AMV reverse transcriptase(BioFlux, Hangzhou, China) was used due to its tolerance to high temperature even at 65 °C. Bst DNA polymerase was changed to Gsp Fast polymerase (Ustar Biotech, Hangzhou, China) to adapt to RNA detection. The optimization condition of all components, except for AMV, Gsp Fast polymerase, and RNA secure, was retained. AMV, Gsp Fast polymerase, and RNA secure in the RT CPA-NATS system were optimized.

### RT CPA-NATS used for detection of PEDV in clinical specimens

We collected 41 viscera samples from pigs apparently infected with PEDV from 3 regions (Chengdu, Harbin, and Jinan) of China to evaluate the PEDV RT CPA-NATS method. Samples 1–20 were obtained from Chengdu, 21–31 from Harbin, and 32–41 from Jinan. We tested the 41 specimens using the constructed PEDV RT CPA-NATS method. In the same manner, RT-PCR tests with outer primers pair of CPA were conducted on these specimens to confirm CPA.

Nonprofessional personnel in a livestock farm conducted the RT CPA-NATS to detect PEDV in the samples to verify the clinical use effect of the RT CPA-NATS method. The same samples were tested using their developed RT-PCR method with the detection primers D-800-F/R, which match M and N gene of PEDV, respectively (Table 1). The product length was 800 bp.

## Additional Information

**How to cite this article**: Wang, F.-X. *et al*. Reverse Transcription Cross-Priming Amplification–Nucleic Acid Test Strip for Rapid Detection of Porcine Epidemic Diarrhea Virus. *Sci. Rep*. **6**, 24702; doi: 10.1038/srep24702 (2016).

## Figures and Tables

**Table 1:** Primers and probes used in CPA-NATS of PEDV.

	Primers name	Primers Sequence(5′-3′)	Labeling terminal and
Primers amplified N gene	N-F	ATGGCTTCTGTCAGCTTTCAGG	
	N-R	TTAATTTCCTGTGTCGAA	
Outer primers	PEDVNgeneF3-1	GGCAACAACAGGTCCAGAT	
	PEDVNgeneB3-1	CGGTCATTTGACTGGTTCC	
Probes	PEDVNgeneDF5F-12	TCAGAACAGAGGAGGCAATA	5′FAM
	PEDVNgeneDF5B1	GAGTAACAACAGAGGCAACAAC	5′BIOTIN
Cross primer	PEDVNgeneCPR-2	AGAGGCAACAACCAGTCC-TTGGACTGGTTACGAGACT	
Detection primer of PEDV	D-800-F	GCTGTGAGTAATCCGAGTT	
	D-800-R	GCTTAGGCTTCTGCTGTT	

**Table 2:** The test results of clinical specimens by RT CPA-NATS and RT-PCR.

	1	2	3	4	5	6	7	8	9	10	11	12	13	14	15	16	17	18	19	20	/
RT CPA-NATS	−	−	−	−	+	−	−	−	−	−	−	−	+	−	−	−	−	−	−	−	/
RT−PCR	−	−	−	−	−	−	−	−	−	−	−	−	−	−	−	−	−	−	−	−	/
21	22	23	24	25	26	27	28	29	30	31	32	33	34	35	36	37	38	39	40	41	
RT CPA−NATS	+	+	+	−	−	−	−	−	−	−	−	+	−	−	+	+	+	+	−	−	−
RT−PCR	+	+	+	−	−	−	−	−	+	−	−	−	−	−	+	−	+	+	−	−	−

**Table 3:** Data from application of the RT CPA-NATS on clinical specimens.

	Positive Number/Sample number		
	Samples from Chengdu	Samples from Harbin	Samples from Jinan
RT CPA-NATS	2/20	3/11	5/10
RT-PCR	0/20	4/11	3/10

## References

[b1] Ojkic D. . The first case of porcine epidemic diarrhea in Canada. Can Vet J 56, 149–152 (2015). 25694663 PMC4298265

[b2] Vui D. T. . Complete genome characterization of porcine epidemic diarrhea virus in Vietnam. Arch Virol 160, 1931–1938 (2015). 26026958 10.1007/s00705-015-2463-6

[b3] Theuns S. . Complete genome sequence of a porcine epidemic diarrhea virus from a novel outbreak in belgium, january 2015. Genome Announc 3, doi: 10.1128/genomeA.00506-15 (2015). PMC4440965 25999551

[b4] Song D. . Molecular characterization and phylogenetic analysis of porcine epidemic diarrhea viruses associated with outbreaks of severe diarrhea in piglets in Jiangxi, China 2013. PLos One 10, e0120310 (2015). 10.1371/journal.pone.0120310 PMC4366183 25790462

[b5] Cho Y. Y. . Complete Genome Sequence of K14JB01, a Novel Variant Strain of Porcine Epidemic Diarrhea Virus in South Korea. Genome Announc 2, doi: 10.1128/genomeA.00505-14 (2014). PMC4038887 24874682

[b6] Sun R. Q. . Outbreak of porcine epidemic diarrhea in suckling piglets, China. Emerg Infect Dis 18, 161–163 (2012). 22261231 10.3201/eid1801.111259 PMC3381683

[b7] Xu G. . Cross priming amplification: mechanism and optimization for isothermal DNA amplification. Sci Rep 2, 246 (2012). 22355758 10.1038/srep00246 PMC3271364

[b8] Fang R. . Cross-priming amplification for rapid detection of Mycobacterium tuberculosis in sputum specimens. J Clin Microbiol 47, 845–847 (2009). 19116359 10.1128/JCM.01528-08 PMC2650920

[b9] Yu X. . Development of a real-time reverse transcription loop-mediated isothermal amplification method for the rapid detection of porcine epidemic diarrhea virus. Virol J 12, 76 (2015). 25972083 10.1186/s12985-015-0297-1 PMC4459462

[b10] Gou H. . Rapid and sensitive detection of porcine epidemic diarrhea virus by reverse transcription loop-mediated isothermal amplification combined with a vertical flow visualization strip. Mol Cell Probes 29, 48–53 (2015). 25444939 10.1016/j.mcp.2014.11.004

[b11] Yuan W. . Development of a nanoparticle-assisted PCR assay for detection of porcine epidemic diarrhea virus. J Virol Methods 220, 18–20 (2015). 25887451 10.1016/j.jviromet.2015.04.008 PMC7113876

[b12] Wang L., Zhang Y. & Byrum B. Development and evaluation of a duplex real-time RT-PCR for detection and differentiation of virulent and variant strains of porcine epidemic diarrhea viruses from the United States. J Virol Methods 207, 154–157 (2014). 25019169 10.1016/j.jviromet.2014.07.005 PMC7113648

[b13] Zhao P. D. . Development of a multiplex TaqMan probe-based real-time PCR for discrimination of variant and classical porcine epidemic diarrhea virus. J Virol Methods 206, 150–155 (2014). 24928691 10.1016/j.jviromet.2014.06.006

[b14] Sozzi E. . Comparison of enzyme-linked immunosorbent assay and RT-PCR for the detection of porcine epidemic diarrhoea virus. Res Vet Sci 88, 166–168 (2010). 19501378 10.1016/j.rvsc.2009.05.009 PMC7111879

[b15] Xiao S. . Simultaneous detection and differentiation of highly virulent and classical Chinese-type isolation of PRRSV by real-time RT-PCR. J Immunol Res 2014, 809656 (2014). 25114934 10.1155/2014/809656 PMC4119655

[b16] Jiang T. . Development of RT-LAMP and real-time RT-PCR assays for the rapid detection of the new duck Tembusu-like BYD virus. Arch Virol 157, 2273–2280 (2012). 22865206 10.1007/s00705-012-1431-7

